# High-frequency oscillations in epilepsy and surgical outcome. A meta-analysis

**DOI:** 10.3389/fnhum.2015.00574

**Published:** 2015-10-20

**Authors:** Yvonne Höller, Raoul Kutil, Lukas Klaffenböck, Aljoscha Thomschewski, Peter M. Höller, Arne C. Bathke, Julia Jacobs, Alexandra C. Taylor, Raffaele Nardone, Eugen Trinka

**Affiliations:** ^1^Department of Neurology, Christian Doppler Medical Centre and Centre for Cognitive Neuroscience, Paracelsus Medical UniversitySalzburg, Austria; ^2^Department of Mathematics, Paris Lodron UniversitySalzburg, Austria; ^3^Department of Neuropediatrics and Muscular Diseases and Epilepsy Center, University Medical CenterFreiburg, Germany; ^4^Department of Neurology, Franz Tappeiner HospitalMerano, Italy

**Keywords:** HFOs, ripples, fast ripples, epilepsy surgery, HFO detection

## Abstract

High frequency oscillations (HFOs) are estimated as a potential marker for epileptogenicity. Current research strives for valid evidence that these HFOs could aid the delineation of the to-be resected area in patients with refractory epilepsy and improve surgical outcomes. In the present meta-analysis, we evaluated the relation between resection of regions from which HFOs can be detected and outcome after epilepsy surgery. We conducted a systematic review of all studies that related the resection of HFO-generating areas to postsurgical outcome. We related the outcome (seizure freedom) to resection ratio, that is, the ratio between the number of channels on which HFOs were detected and, among these, the number of channels that were inside the resected area. We compared the resection ratio between seizure free and not seizure free patients. In total, 11 studies were included. In 10 studies, ripples (80–200 Hz) were analyzed, and in 7 studies, fast ripples (>200 Hz) were studied. We found comparable differences (dif) and largely overlapping confidence intervals (CI) in resection ratios between outcome groups for ripples (dif = 0.18; CI: 0.10–0.27) and fast ripples (dif = 0.17; CI: 0.01–0.33). Subgroup analysis showed that automated detection (dif = 0.22; CI: 0.03–0.41) was comparable to visual detection (dif = 0.17; CI: 0.08–0.27). Considering frequency of HFOs (dif = 0.24; CI: 0.09–0.38) was related more strongly to outcome than considering each electrode that was showing HFOs (dif = 0.15; CI = 0.03–0.27). The effect sizes found in the meta-analysis are small but significant. Automated detection and application of a detection threshold in order to detect channels with a frequent occurrence of HFOs is important to yield a marker that could be useful in presurgical evaluation. In order to compare studies with different methodological approaches, detailed and standardized reporting is warranted.

## 1. Introduction

For epilepsy patients not responding to medication, surgery is the most important treatment option with a realistic hope of seizure freedom (Wiebe et al., 2001; Wieser et al., 2001; McIntosh et al., 2004; Wellmer et al., 2012). The surgical intervention aims at removing or disconnecting the entire epileptogenic zone, defined as the region which is indispensable for generating seizures (Rosenow and Lüders, 2001). As a consequence, up to 60–80% of operated patients will achieve seizure freedom (Wiebe et al., 2001; Engel et al., 2012; Schulze-Bonhage and Zentner, 2014). For successful surgical treatment it is critical to determine the epileptogenic zone as precisely as possible. However, no diagnostic modality available today unambiguously delineates the epileptogenic zone (Rosenow and Lüders, 2001).

Throughout the last decade, increasing attention has been paid to fast (250–500 Hz) and ultrafast (>500 Hz) EEG oscillations as a measurable electrophysiological component of potentially pathological brain activity (Allen et al., 1992; Fried et al., 1999; Jacobs et al., 2012). Despite a continuously evolving understanding of the importance of high-frequency activity in the physiological context, such as memory consolidation, alertness and arousal (Buzsáki and Lopes da Silva, 2012), fast and ultrafast EEG activity is also considered to be a promising marker of epileptogenicity (Bragin et al., 1999; Zijlmans et al., 2012). It should be noted that there is no precise specification of high-frequency oscillations (HFO). The term subsumes activity in frequencies above 80 or 100 Hz, physiological as well as pathological, and of diverse origin.

Interest in HFOs has been kindled by their potential use as a biomarker for epileptogenic brain tissue. Recent studies revealed that resection of areas identified as generating (pathological) oscillations well beyond 80 Hz leads to excellent postsurgical outcomes (Jacobs et al., 2010a,b, 2012). It was discovered that pathological interictal HFOs delineate the seizure onset zone to a large extent independent of spikes, specifically those in higher frequency bands, such as the fast ripples in the range of 250–500 Hz (Jacobs et al., 2008), and more reliably than the underlying presumed epileptogenic lesion, especially, when their localization is discordant to the spike localization or the seizure onset zone (Jacobs et al., 2009). High-frequency oscillations were less sensitive but much more specific and accurate than epileptic spikes in delineating the seizure onset zone (Andrade-Valenca et al., 2011).

Despite there being some promising results, a Cochrane review concluded that the evidence for effective use of HFOs for epilepsy surgery decision-making is rather poor (Gloss et al., 2014). Thus, the application of HFOs for pre-surgical evaluation is in an early stage of development and further research is highly warranted.

### 1.1. Pre-surgical evaluation of epilepsy

**Figure 1:**
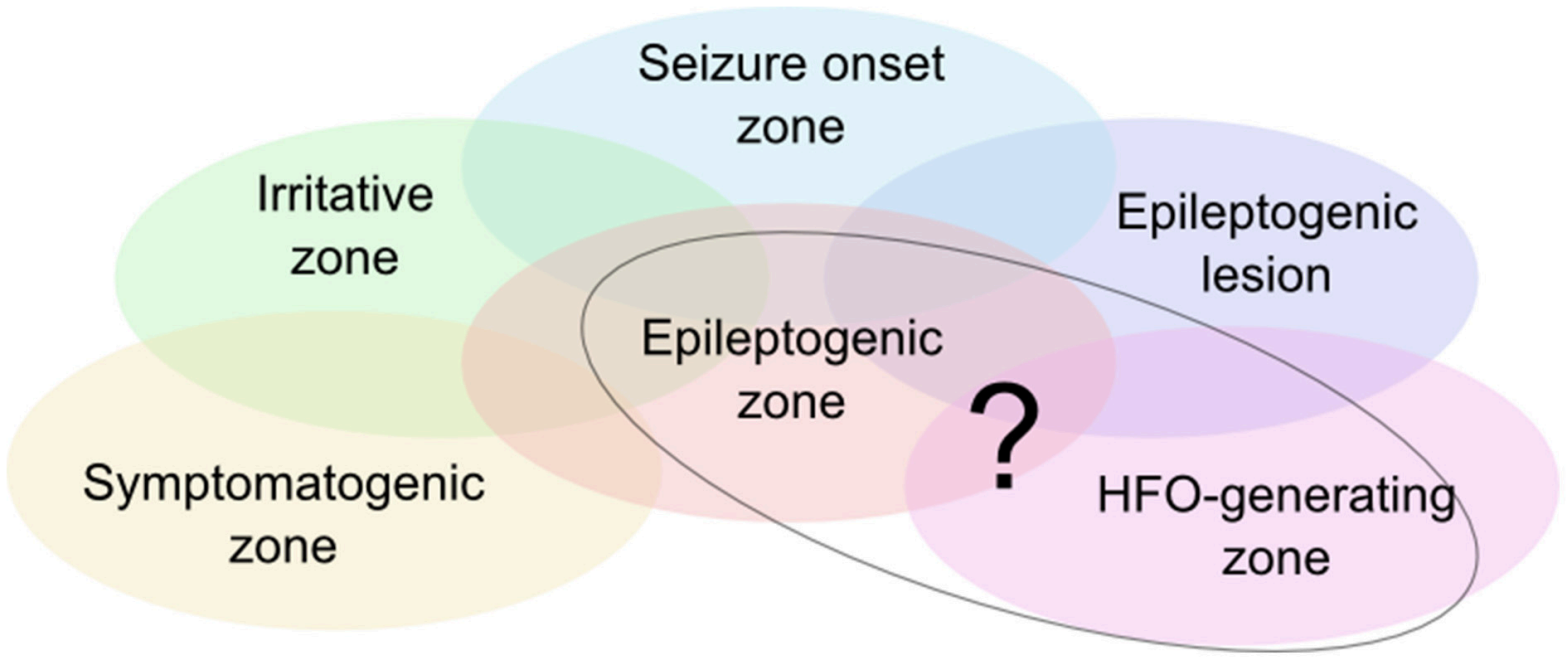
**Illustration of the zone model as defined by Rosenow and Lüders (2001)**. There are currently no diagnostic means to unambiguously delineate the epileptogenic zone, so that it needs to be estimated based on various diagnostic measures. HFOs are considered a further piece of information to circumscribe the epileptogenic zone. Ongoing research assesses the degree of correlation of the HFO-generating zone with the other zones, in particular the epileptogenic zone.

The epileptogenic zone is one of the key concepts of surgical treatment of epilepsy patients (see Figure 1, Rosenow and Lüders, 2001). It is defined as covering exactly those regions of the cortex that have the actual or potential capability of inducing seizures and that should ideally be removed completely in the surgical process. The post-surgical outcome therefore depends heavily upon the precision and accuracy in defining and resecting these areas.

Due to a lack of tools and methods which measure and define the epileptogenic zone directly, this region is assessed indirectly on the basis of mutually complementing diagnostic approaches and a resulting set of specific zones built upon measurable or observable parameters (Kilpatrick et al., 2003; Asano et al., 2013; Fernández and Loddenkemper, 2013).

Today it is understood that no single one of the other zones (see Figure 1) directly reflects the epileptogenic zone, spurring research for new and potentially more precise biomarkers. Interictal HFOs could eventually permit a more direct definition of the epileptogenic zone (Jacobs et al., 2012; Fernández and Loddenkemper, 2013).

### 1.2. HFOs in the pre-surgical evaluation of epilepsy

Soon after recognizing the potential of HFOs to identify the epileptogenic zone (Bragin et al., 1999), studies were designed to determine the meaning and relevance of HFOs in the presurgical evaluation of refractory epilepsy. Similar to the findings of Bragin et al. (2002) in the entorhinal cortex, Staba et al. (2002) reported an increased ratio of fast ripple (250–500 Hz) to ripple (100–200 Hz) activity in the hippocampus and entorhinal cortex ipsilateral to seizure onset compared to the contralateral site. The finding that interictal HFOs were consistent with the seizure onset zone has been replicated several times (Blanco et al., 2011; Andrade-Valença et al., 2012; Cho et al., 2012).

HFOs have been found to be related to medication effects, seizure activity, and seizure severity (Zijlmans et al., 2009a,b). It has also been suggested that HFOs are a reliable marker for focal cortical dysplasia (Kerber et al., 2013). In addition, HFOs occur more frequently at seizure onset (Pearce et al., 2013; Perucca et al., 2014) and have been used to document functional isolation of the epileptogenic zone in the interictal state (Van Diessen et al., 2013). Compared to interictal spikes, ictal HFOs correlate more strongly with seizures and have a more stable localization (Zijlmans et al., 2011), but HFO rates can be modulated by stages of the sleep-wake cycle, depending on the region of interest (Dümpelmann et al., 2015). Several studies have shown that the complete removal of HFO-generating areas is an indicator of good surgical outcome (Jacobs et al., 2010b; Wu et al., 2010; Akiyama et al., 2011; Modur et al., 2011; Fujiwara et al., 2012; Hägelen et al., 2013; Cho et al., 2014; Kerber et al., 2014a; Weiss et al., 2014).

There is reasonable evidence that HFOs are a promising biomarker for epileptogenicity and ictogenesis (Worrell and Gotman, 2011; Jacobs et al., 2012; Jette and Wiebe, 2013; Staba et al., 2014). Contradicting opinions raise doubts about the clinical significance of HFOs, since the relationship to the epileptic process is not understood in detail (Jobst, 2013). In a recent Cochrane review (Gloss et al., 2014), among 34 potentially relevant articles, only two met the restrictive inclusion criteria. These two were the only prospective studies, and in total, only 11 patients were examined.

Prospective studies on HFOs, albeit essential, may not be encouraged by this result (Ibrahim et al., 2012). Still, despite the potential bias of retrospective studies, it is worth evaluating these data before planning a prospective study. Major problems in gathering information from retrospective studies on resection of HFO-generating tissue are the heterogeneity of these studies in clinical and statistical respects, as well as differences in reporting the results. A systematic review may count the number of studies with evidence for and against removal of HFO-generating tissue, but only a meta-analysis is able to synthesize data from a series of separate studies. In other words, a Cochrane review (Gloss et al., 2014) could not draw any conclusions because the level of evidence is at an early stage; but a meta-analysis could better characterize the state of research, forming the base for future clinical studies. By conducting a meta-analysis, we would like to

1. statistically characterize the effect with a greater power and higher precision (smaller confidence-interval) than single studies,
2. assess heterogeneity of results across studies,
3. identify moderators that explain variation between studies,
4. examine the presence of publication bias,
5. emphasize the importance of detailed and standardized reporting of single-patient data and propose a checklist for studies on HFOs in pre-surgical decision making.

## 2. Materials and methods

### 2.1. Data sources

We performed an electronic literature search in pubmed, Cochrane databases, and medline (accessed through Ovid) from inception to the 5th February, 2015 by using the following terms:

- high frequency oscillations OR ripple OR fast oscillations AND
- epilepsy OR seizure AND
- surgery OR resection

Additionally, the bibliography of the identified articles was searched manually in order to retrieve additional literature.

### 2.2. Inclusion criteria

We included articles if they met all of the following criteria:

1. reporting of ictal or channel-wise interictal HFO occurrence and outcome after surgery, AND
2. written in English or German and full text available, AND
3. human participants, AND
4. outcome according to Engel (Engel et al., 1993) or ILAE (Wieser et al., 2001) classification or a classification into seizure free vs. non-seizure free AND
5. reporting of number of channels on which HFOs were detected and, among these, number of channels which were inside the resected area OR authors made this information available upon request.

### 2.3. Data extraction

We extracted the following information:

1. reporting of number of channels on which HFOs were detected and, among these, number of channels which were inside the resected area
2. outcome for each patient and time of follow up
3. are results based on ripples (80–200 Hz) or fast ripples (>200 Hz)
4. was it an adult or pediatric population
5. were HFOs recorded pre-, peri-, post-ictal, or ictal
6. were HFOs recorded intracranially or from the surface; and for intracranial recordings:
  - were depth-electrodes or subdural grids used
  - what size were the electrodes
7. were channels judged to show HFOs at all, or were channels rated based on the occurrence of HFOs within a certain time-interval (i.e., a threshold was determined to distinguish high- vs. low frequency of HFO-occurrence on a channel)
8. were HFOs detected visually or based solely on an automated detector.

Three reviewers independently performed the data extraction, two (YH and PH) in pubmed and two (YH and AT) in the Cochrane databases and medline. Any discrepancies were resolved via discussion, to include only data with mutual agreement.

### 2.4. Data classification

We calculated the resection ratio by dividing the number of channels on which HFOs were detected and which were inside the resected area by the total number of channels on which HFOs were detected.

We formed groups of patients for each study according to outcome. We compared seizure free patients, having an outcome of Engel class Ia or ILAE I, and non-seizure free patients, being equivalent to Engel class Ib-IV and ILAE II-VI (including auras).

In two publications there was no obvious distinction between Engel classes Ia vs. rest since the subgrouping of a, b, …etc. was not added to the outcome class and the description did not directly state that class I would be equivalent to seizure freedom. This information is missing in Okanishi et al. (2014) and Modur et al. (2011) in 3 patients in each of the two studies. We considered these patients as seizure free (class Ia).

### 2.5. Data analysis

As an effect size *z* we calculated the difference of the average resection ratio for the group of patients with good and bad outcome:

$$\begin{array}{l} z={\hat{\mu }}_{sf}-{\hat{\mu }}_{nsf}, \end{array}$$

where *sf* indicates the seizure free group, *nsf* indicates the non-seizure free group, and ${\hat{\mu }}_{x}$ is the average resection ratio within the group specified by the respective subindex. The variance *s*^2^ of *z* was estimated as

$$\begin{array}{l} s^{2}=\frac{1}{n_{sf}}\cdot V_{sf}+\frac{1}{n_{nsf}}\cdot V_{nsf}, \end{array}$$

where *n*_*x*_ is the number of patients within the specified outcome group, and *V*_*x*_ is the estimated within-group variance, calculated as

$$\begin{array}{l} V_{x}=\frac{1}{n_{x}-1}\cdot \overset{n_{x}}{\underset{i}{\sum }}{\left(X_{xi}-{\hat{\mu }}_{x}\right)}^{2}, \end{array}$$

denoting by *X*_*xi*_ the individual value observed on patient *i* in group *x* (where *x* is either *sf* or *nsf*).

In one study (Jacobs et al., 2010b), there was only one seizure free patient. Therefore, we chose a conservative calculation model of the variance. We calculated *V*_*x*_ for the whole group (good and bad outcome) and used this as an estimate for *s*^2^.

We used the R package *metafor* (Viechtbauer, 2010) for further analysis. A random effect model was fitted by estimating the amount of heterogeneity by the DerSimonian-Laird estimator (DerSimonian and Kacker, 2007).

The analysis was conducted separately for ripples and fast ripples. In addition, we grouped the studies into those which performed an automated and those which performed a visual detection of HFOs, and into those in which channels were only considered as containing HFOs if the rate of HFO-occurrence/time interval exceeded a defined threshold and those which did not use such a threshold. This was only done for ripples, because the subgroups for fast ripples would have been too small.

Patients in which neither ripples nor fast ripples were found were excluded from the respective analysis.

### 2.6. Analysis of publication bias

To analyze a possible bias of publication, we built funnel plots and applied the trim and fill method as proposed by Duval and Tweedie (2000a,b). The method estimates the number of studies missing from a meta-analysis due to unpublished results, in our case with no or negative relation between resection ratio and outcome. The estimated number of studies is then added to the available studies, making the funnel plot more symmetric. The estimated new outcome can show what effect would be observable if there were really unpublished studies with null or negative effects.

## 3. Results

### 3.1. Studies included in the meta analysis

**Figure 2:**
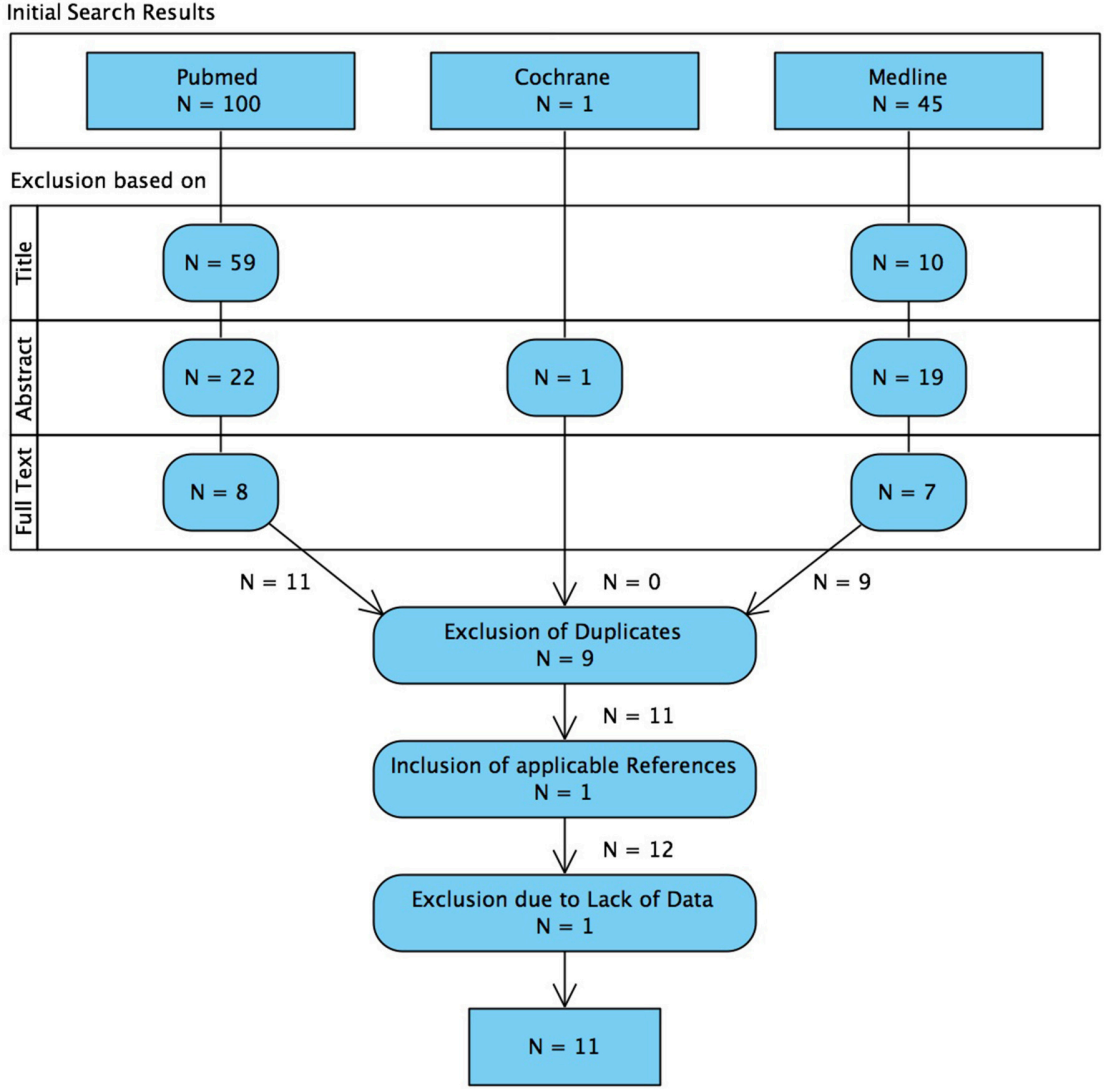
**Flowchart illustrating the systematic search of literature and the selection process of articles**. We want to mention that 4 articles were considered to be relevant, but excluded already on the basis of the whole text because of non-quantitative reporting of results (Ramachandrannair et al., 2008; Wu et al., 2010; Nariai et al., 2011; Iwatani et al., 2012).

The search and exclusion of articles is illustrated in Figure 2 and for the identified articles, the methodology is listed in Table 1, the details on the samples in Table 2, and the recording techniques in Table 3.

**Table 1:** **Methodological details of the identified articles**.

**Study**	**Ripple, fast ripple**	**Sampling rate (kHz)**	**Time**	**Threshold**	**Detection**	**Outcome**	**Follow-up**
Ochi et al., 2007	R	1	pre-/ ictal	No	Visual	Engel	17.4 (11–23)
Jacobs et al., 2010b	R, FR	2	SWS	No	Visual	Engel	22.7 (6.8)
Akiyama et al., 2011	R, FR	1	NREM	Yes	Auto	ILAE	24
van't Klooster et al., 2011	R, FR	2	e-stim	Yes	Visual	Engel	>12
Modur et al., 2011	R	1	ictal	Yes	Visual	Engel	26.5 (6.57)
Usui et al., 2011	FR	1	inter-/ ictal	No	Visual	Engel	12–54
Fujiwara et al., 2012	R (FR)	2	ictal	No	Visual	Seizure free	n.a.
Cho et al., 2014	R, FR	2	SWS	Yes	Auto	Engel	26.33 (4.76)
Kerber et al., 2014b	R (FR)	1	SWS	No	Visual	Engel	52.94 (18.27)
van Klink et al., 2014	R, FR	2	io	No	Auto	Engel	12
Okanishi et al., 2014	R, FR	1–2	NREM	Yes	Auto	Engel	58 (19–76)

The columns indicate whether R, Ripples; and/or FR, Fast Ripples were assessed, the kind of EEG/the time when HFOs were searched in the EEG; i.e., during SWS, interictal slow wave sleep; NREM, interictal non-rapid eye movement sleep; io, intra-operative EEG; or during e-stim, electrical stimulation; further, the columns indicate whether the study used a threshold (thresh) so that channels were considered as containing HFOs only if the rate of HFO-occurrence/time interval exceeded a defined threshold, whether the study used auto: automated, or visual detection of HFOs, and which outcome classification was used. Finally, follow-up is indicated as general follow-up time for all patients or in average months along and/or with the range or standard deviation, depending on what information was given in the original paper. n.a., not available.

**Table 2:** **Patient details of the identified articles**.

**Study**	**Age**	**Number seizure free patients**	**Number non seizure free patients**
Ochi et al., 2007	4–17	4	5
Jacobs et al., 2010b	21–55	1	19
Akiyama et al., 2011	1–18	10	18
van't Klooster et al., 2011	8–42	4	5
Modur et al., 2011	6–30	3	3
Usui et al., 2011	1–27	4	7
Fujiwara et al., 2012	0.75–25	23	18
Cho et al., 2014	12–44	10	5
Kerber et al., 2014b	8–47	13	3
van Klink et al., 2014	3–37	7	7
Okanishi et al., 2014	3–18	3	7

Patient details with age range in years and number of patients in the two outcome groups (seizure free vs. non-seizure free).

**Table 3:** **Recording techniques in the identified articles**.

		**ECoG recordings**			**Depth electrodes**		
**Study**	**Recording**	**Diameter**	**Exposure**	**Effective surface**	**Diameter**	**Surface**	**Length**
Ochi et al., 2007	sd ECoG	4 mm			–	–	–
Jacobs et al., 2010b	depth	–	–	–		0.8 mm^2^	
Akiyama et al., 2011	sd ECoG, depth	4 mm	2.3 mm^2^	4.2 mm^2^		8.3 mm^2^	
van't Klooster et al., 2011	sd ECoG, depth			4.2 mm^2^		7.9 mm^2^	
Modur et al., 2011	sd ECoG, depth		2.3 mm^2^			1.1 mm^2^	
Usui et al., 2011	sd ECoG, depth		2.3 mm^2^	4.15 mm^2^	0.8 mm		1 mm
Fujiwara et al., 2012	sd ECoG		< 2.5 mm^2^		–	–	–
Cho et al., 2014	sd ECoG, depth	4 mm	2.3 mm^2^				2.3 mm
Kerber et al., 2014b	sd ECoG		2.3 mm^2^		–	–	–
van Klink et al., 2014	io ECoG	n.a.	n.a.	n.a.	–	–	–
Okanishi et al., 2014	sd ECoG, depth	4 mm	2.3 mm^2^	4.2 mm^2^		8.3 mm^2^	

Recording techniques including sampling rate, method being sd, subdural; io, intra-operative, ECOG, electrocorticogram n.a., not available, and characteristics of the electrodes.

It is worth mentioning that 4 articles were considered to be relevant, but excluded already on the basis of the whole text because of non-quantitative reporting of results. The studies of Ramachandrannair et al. (2008), Wu et al. (2010), Nariai et al. (2011), and Iwatani et al. (2012) reported HFO-detection not in a channel-wise manner but named the regions where the HFOs were detected. Accordingly, these studies listed the removed regions. This does not allow for quantitative analyses, but we will include the results of these studies into the discussion of our study.

Finally, the work of Hägelen et al. (2013) was not included because 20 of the 30 patients were already included in the work of Jacobs et al. (2010b) and, additionally, they did not report on the occurrence of HFOs on channels but calculated the average number of HFOs per minute of resected and non-resected tissue and thereof the ratio.

Although in the study of Jacobs et al. (2010b) determined the HFO-rate for each channel in their study, the resection ratio was calculated based on the overall occurrence of HFOs, which was also used for our meta-analysis. Modur et al. (2011) did not calculate a rate, but used a similar concept: only electrodes which showed HFOs that evolved over the course of the seizure were considered as channels with HFOs.

The publication of van Klink et al. (2014) only reported results based on the HFO-rate. We were able to include this work into the meta-analysis because the first author supported us by providing the information on the resection ratio.

Some studies assessed ripples and fast ripples separately, but some did not distinguish between the frequency bands. Fujiwara et al. (2012) defined HFOs between 80 and 500 Hz. We only included the results of this study in the ripple-analysis. Kerber et al. (2014b) reported results for ripples and fast ripples, but the detailed data which were usable for our meta-analysis were based on ripples, only.

All of the identified studies were based on intracranial recordings. The technical details of the recordings/electrodes are listed in Table 3.

### 3.2. Outcome and ripple-resection ratio

**Figure 3:**
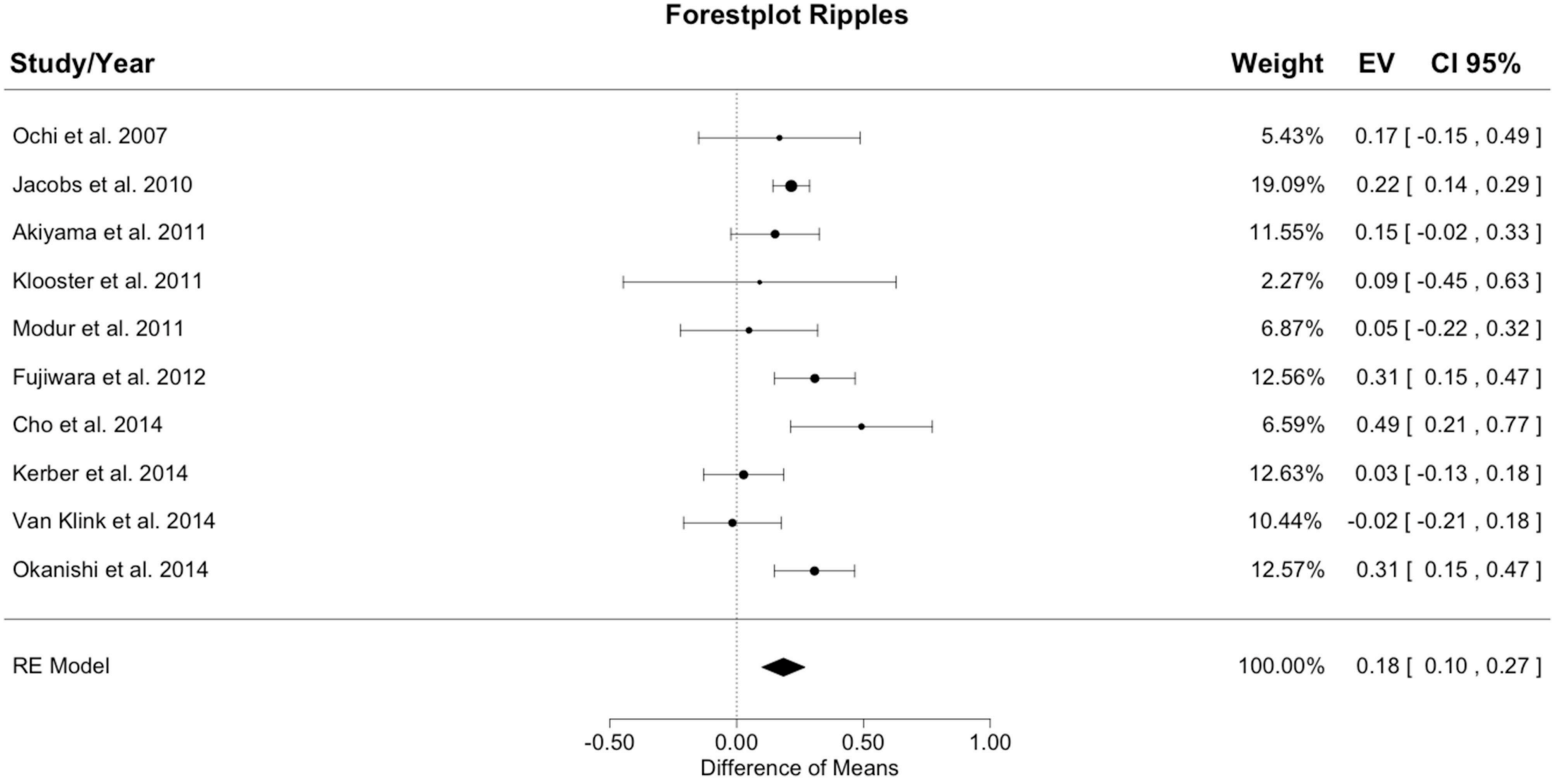
**Meta-analysis results for ripples**. The resection ratio for ripples is higher in seizure free patients compared to non-seizure free patients. For each study, a graphical representation of the effect (i.e., the difference of the resection ratio between the good- and bad-outcome group) and of the confidence interval (CI) is given along with the exact values (EV) and the weights.

Figure 3 shows the meta-analysis result for ripples. The random effects model yielded a total heterogeneity with *I*^2^ = 52.81% and *Q*(*df* = 9) = 19.07; *p* = 0.025. The estimated model result was significant with a difference of resection ratios between groups of 0.184 (*SE* = 0.044; *z* = 4.22; *p* < 0.001).

The resection ratio was higher in seizure free patients than in non-seizure free patients in 9 out of 10 studies, but in 5 of the 9 positive studies the confidence intervals (CIs) overlapped with 0. Therefore, only 4 studies can be considered to indicate a significantly higher resection rate in seizure free patients compared to non-seizure free patients. Pooling all results in the meta-analysis yielded a positive difference without overlap with 0, so that in general, it could be concluded that the resection ratio for ripples is higher in seizure free patients compared to non-seizure free patients. Nevertheless, the lower bound of the CI is very close to zero, indicating a rather small effect.

### 3.3. Outcome and fast ripple-resection ratio

**Figure 4:**
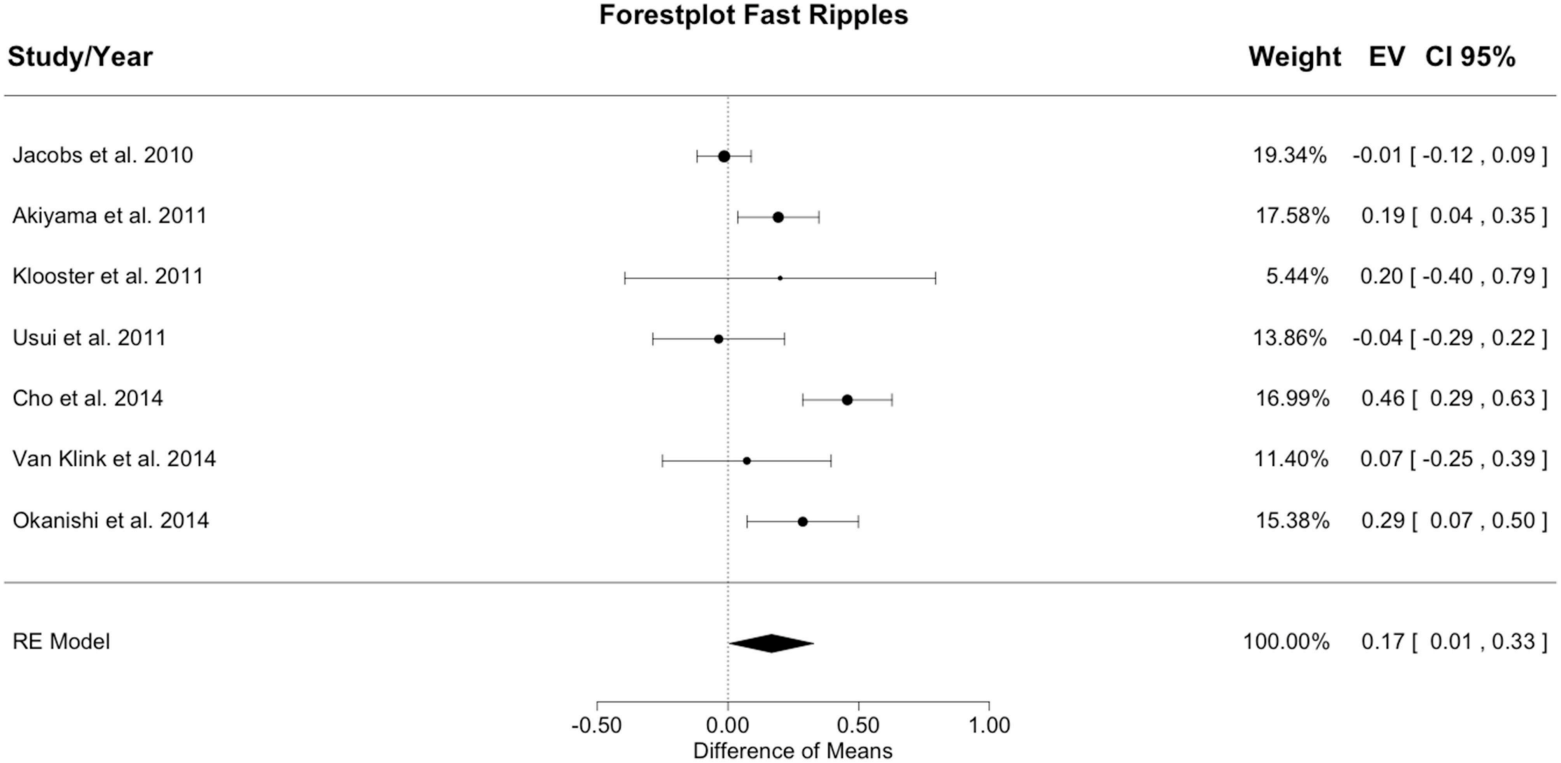
**Meta-analysis results for fast ripples**. The resection ratio is higher in seizure free patients compared to non-seizure free patients. For each study, a graphical representation of the effect (i.e., the difference of the resection ratio between the good- and bad-outcome group) and of the confidence interval (CI) is given along with the exact values (EV) and the weights.

Figure 4 shows the meta-analysis result for fast ripples. The random effects model yielded a total heterogeneity with *I*^2^ = 77.01% and *Q*(*df* = 6) = 26.101; *p* < 0.001. The estimated model result was significant with a difference of resection ratios between groups of 0.167 (*SE* = 0.082; *z* = 2.028; *p* = 0.042).

Resection ratio was higher in seizure free compared to non-seizure free patients in 5 out of 7 studies, but in 2 studies the CIs overlapped with 0. Therefore, only 3 studies can be considered to indicate a significantly higher resection rate in seizure free patients compared to non-seizure free patients. Pooling all results in the meta-analysis yielded a positive difference without overlap with 0, so that in general, it could be concluded that the resection ratio for fast ripples is higher in seizure free patients compared to non-seizure free patients. The effect size for fast ripples is slightly smaller than that for ripples, but the CI is broader and largely overlaps with the CI of the result for ripples. Thus, speculations about differences between ripples and fast ripples are not supported by the data.

### 3.4. Automated vs. visual detection of HFOs

For visual detection, the random effects model yielded a total heterogeneity with *I*^2^ = 36.54% and *Q*(*df* = 5) = 7.879; *p* = 0.163. The estimated model result was significant with a difference of resection ratios between groups of 0.172 (*SE* = 0.048; *z* = 3.589; *p* < 0.001; *CI* = 0.08−0.27). For automated detection, the random effects model yielded a total heterogeneity with *I*^2^ = 73.07% and *Q*(*df* = 3) = 11.14; *p* = 0.011. The estimated model result was significant with a difference of resection ratios between groups of 0.219 (*SE* = 0.096; *z* = 2.288; *p* = 0.022; *CI* = 0.03−0.41).

The effect for automated detection is comparable to visual detection, but again the CIs do completely overlap. The analysis was not done for fast ripples but it should be noted that 4 out of 7 studies which examined fast ripples used automated detection. In contrast, only 2 out of 10 studies which examined ripples used automated detection.

Supplementary Figure 1 shows the meta-analysis result for ripples that were detected visually and supplementary Figure 2 shows the meta-analysis results for ripples that were detected automatically.

### 3.5. Thresholding

For detection without a threshold, the random effects model yielded a total heterogeneity with *I*^2^ = 63.82% and *Q*(*df* = 4) = 11.06; *p* = 0.026. The estimated model result was significant with a difference of resection ratios between groups of 0.149 (*SE* = 0.06; *z* = 2.492; *p* = 0.013; *CI* = 0.03−0.27). For detection based on a threshold, the random effects model yielded a total heterogeneity with *I*^2^ = 43.09% and *Q*(*df* = 4) = 7.029; *p* = 0.134. The estimated model result was significant with a difference of resection ratios between groups of 0.235 (*SE* = 0.073; *z* = 3.228; *p* = 0.001; *CI* = 0.09−0.38).

Studies using a threshold considered channels as showing HFOs only if the occurrence of HFOs exceeded a defined threshold (usually high occurrence rate in time). It seems that the difference between outcome groups is slightly higher in those studies which applied a threshold compared to those which did not. Again, the analysis was not done for fast ripples. There were 4 out of 7 studies which used a threshold for examining fast ripples, while 5 out of 10 studies which examined ripples used a threshold.

Supplementary Figures 3, 4 show the meta-analysis result for studies which did not or did use a threshold to assess the occurrence of ripples, respectively.

### 3.6. Analysis of publication bias

**Figure 5:**
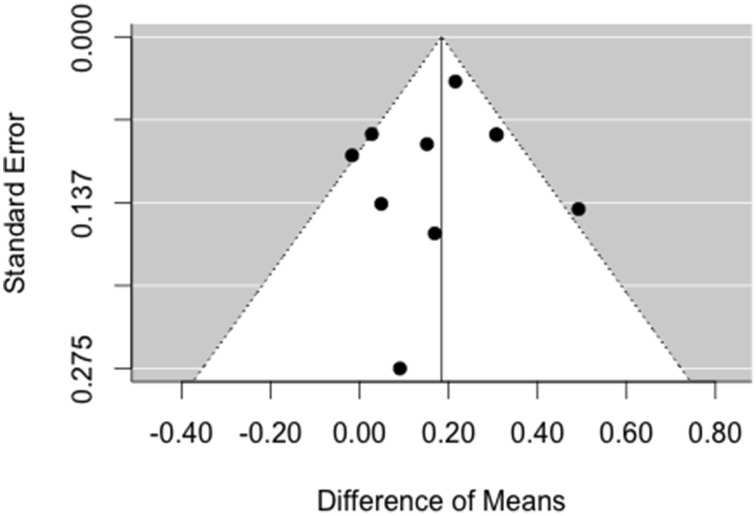
**Funnel plot for analysis of publication bias for ripples**. There is no asymmetry pointing toward missing studies on the left side of the plot.

As can be seen in Figure 5, the result asymmetry of the funnel plot is rather due to a lower number of studies on the right side, i.e., there are more studies with small, null, and negative effects than studies with a large effect. Accordingly, the trim and fill method does not augment the data, because the model assumes that there is no publication bias on the left side.

**Figure 6:**
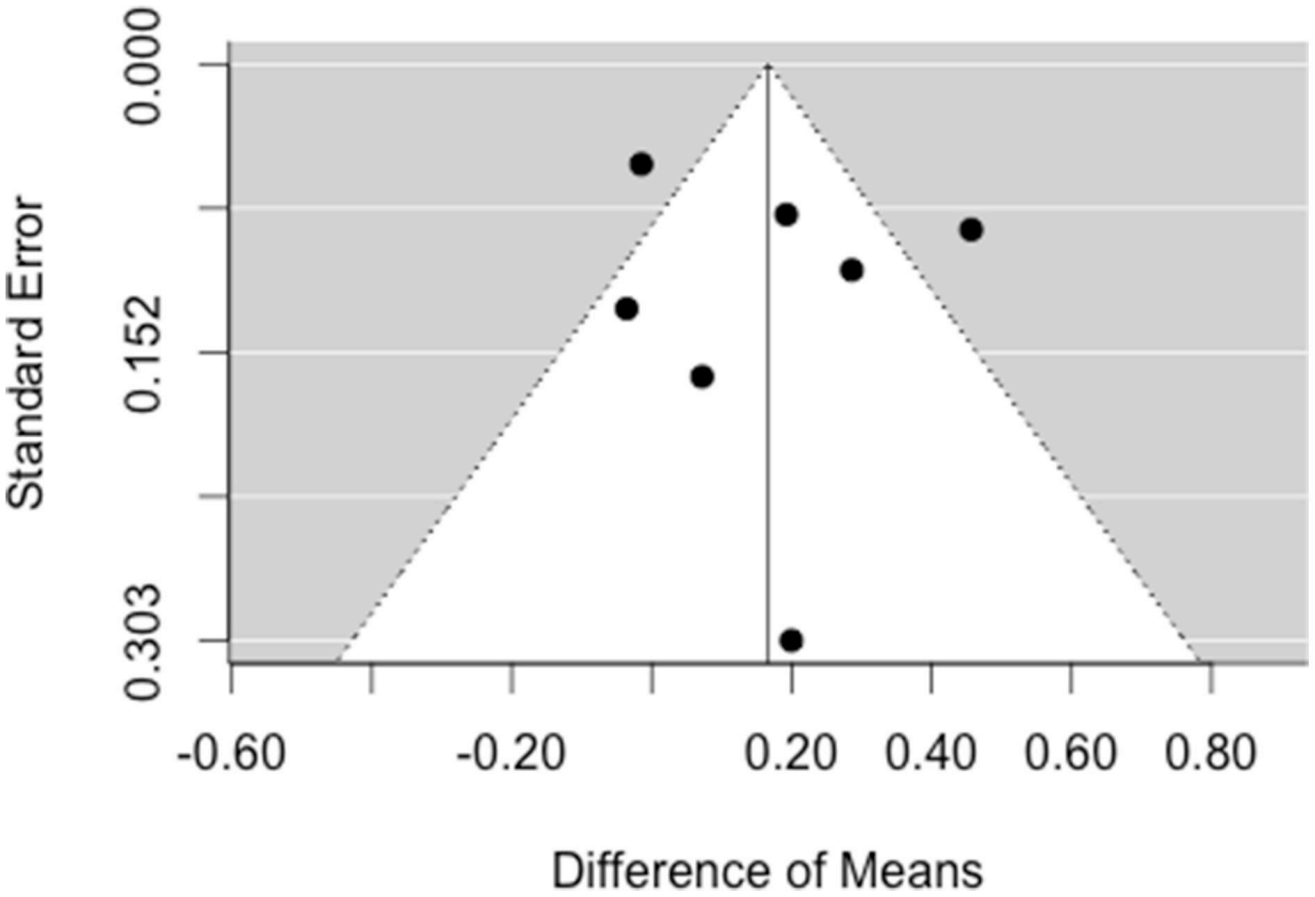
**Funnel plot for analysis of publication bias for fast ripples**. There is no asymmetry pointing toward missing studies on the left side of the plot.

For fast ripples, there is no asymmetry of the funnel plot in Figure 6 on the left side. Again, the trim and fill method does not augment the data, because the model assumes that there is no publication bias.

## 4. Discussion

In the discussion, we aim to address the five goals which we defined in the introduction.

### 4.1. Characterization of the effect

This meta-analysis confirmed a higher resection ratio for HFOs in seizure free patients vs. non-seizure free patients. Nevertheless, the effect is rather small with a mean difference between outcome groups of 0.18 and 0.17 in the resection ratios of ripples and fast ripples, respectively.

Both types of HFOs show a higher resection rate in seizure free patients compared to non-seizure free patients. The difference between these groups was slightly larger for ripples, but the variance does not allow any conclusions to be drawn. An important weakness of the analysis is that ripples and fast ripples were mixed in Fujiwara et al. (2012) and we included the result in the ripple-class. This may have lead to an indistinguishable effect. Moving this study from the ripple to the fast-ripple analysis would decrease the effect of ripples and increase that of fast ripples, but the difference between ripples and fast-ripples would still lack of significance (i.e., the confidence intervals would still overlap completely).

The evidence for fast ripples is low in terms of studies and sample sizes. This is possibly due to the difficulty in properly detecting oscillations above 200 Hz. Classical sampling rates do not allow the detection of fast ripples, at all. Micro-electrodes are thought to better capture fast ripples because of local propagation characteristics in this frequency range (Worrell et al., 2008). Almost all results in our study were based on macro-electrodes, so that further outcome-oriented studies with micro-electrodes are warranted.

At this point it is important to note that, in mathematical terms, all we know is *P(high resection ratio*|*seizure free)*. That is, we characterized the probability that a patient, who is seizure free after surgical intervention, had a high resection ratio. However, the probability *P(seizure free*|*high resection ratio)* might be of greater interest. That is, we would like to know how likely it is that a patient will become seizure free if the resection ratio is high. As discussed by Gloss et al. (2014), the evidence for this latter concept is low and remains to be established by future research.

#### 4.1.1. Excluded articles

There were 4 studies which could not be included into the meta-analysis because the authors applied a qualitative evaluation.

Ramachandrannair et al. (2008) studied the relation between ictal HFOs and spasms in 5 children. The authors found that those 3 patients with complete resection of the HFO-generating area were seizure free, while the other 2 patients with partial removal experienced a reduction of seizures.

Wu et al. (2010) performed intra-operative ECoG in 30 children. Out of these, 24 showed fast ripples (240–500 Hz). In 20 children, the whole HFO generating area was resected, in 3 patients the resection involved only part of the HFO generating area and in one patient, the area was not resected. All but one of the children with a complete resection became seizure free, while this was not the case in the other 4 children.

Nariai et al. (2011) assessed the relation between ictal HFOs and spasms in 11 children. They were able to show that complete resection of the region where HFOs increased significantly at seizure onset was more likely to result in seizure freedom than incomplete resection.

Iwatani et al. (2012) examined four children with symptomatic West syndrome and analyzed HFOs in ictal EEG in relation to spasms. In two of these children, surgery was performed and resulted in seizure freedom. The resection included those regions where ripple-band HFOs were most prominent.

Although these results sound encouraging, we strongly recommend that future studies conform to a common reporting style, which allows objective comparison of results across studies.

It is important to note that all of the excluded articles were based on children. This could be a source of bias, since resection in children is typically larger than in adults. If effects of removal of HFO generating tissue in children-based studies are large, this might be biased by the effect of better outcome with larger resection. Therefore, excluding these articles may not necessarily bias the result of the meta-analysis.

### 4.2. Heterogeneity

Heterogeneity across studies was substantial for ripples and considerable for fast ripples. Indeed, this quantitative characterization of inconsistency is not surprising when reviewing the differences in methodology across groups. By discussing the sources of heterogeneity, we want to emphasize the need for standards in this field of research.

Tables 1–3 showed details and, thus, differences between studies in the examined population (children/adults), the time from which HFOs were extracted (pre-/peri-/post-ictal, intra-operative, or under stimulation), the detection mode (visual vs. automated), application of a threshold to consider a channel to be showing HFOs or not, the time of follow-up, and the electrodes.

First, we believe that it is necessary to distinguish between HFOs in children and adults, but subgroup analyses were not possible because detailed information on single patients was missing in several studies.

Second, the condition (called time in Table 1) may be an important source of inconsistency. Several studies used EEG segments from sleep stages, but a recent study claims that HFOs occur independently from the sleep-wake cycle (Dümpelmann et al., 2015). Accordingly, analyzing interictal EEG should yield similar results. From the selected studies, 4 assessed HFOs in the ictal EEG. When assessing ictal EEG, a new source of variance is present: the type of the seizure. According to Ochi et al. (2007), the type of the seizure moderates the occurrence of HFOs. Therefore, rather than contrasting HFOs in ictal vs. interictal EEG, one should contrast HFOs in ictal EEG of different seizures. Gathering this information for a meta-analysis was not possible with the present studies.

Third, outcome reporting (in short: Engel vs. ILAE classification) is somewhat heterogeneous between studies, but it is still possible to harmonize it to a certain extent. Two studies (Modur et al., 2011; Okanishi et al., 2014) did not distinguish between Engel class 1a vs. 1b etc., but since the number of patients which could potentially be misclassified is very small (in total: 6 patients) this should not largely affect the results. Moreover, the definition of seizure freedom depends largely on the seizure frequency before the intervention, and thus accurate recording or reporting of seizures and a long follow-up period are needed. Information on seizure frequency was not available in the studies. Consequently, this bias could not be handled by the meta-analysis, but this should not systematically affect the result.

Fourth, as can be seen in Table 3, the information given on the electrodes used is not always complete and when extracting these data, we encountered some ambiguous situations. It is highly likely, that at least the recording techniques for subdural ECoG were comparable across studies. In contrast, depth electrodes may vary between studies. It is of interest that only one study (Jacobs et al., 2010b) used a recording technique which is oriented at the classical depth electrodes, but the small recording surface is similar to micro- electrodes. Thus, most studies used macro-electrodes, although use of micro-electrodes is recommendable when the study aims at the detection of fast ripples (Worrell et al., 2008). The use of micro-electrodes is limited to multielectrode arrays or micro-wire bundles for mesial structures (Misra et al., 2014). Because of their low spatial coverage, bundles are not useful for evaluating HFOs in relation to the epileptogenic zone. The use of radially oriented microwires along the shaft of the depth electrodes still needs to be established in HFO research (Worrell et al., 2008).

In addition, Table 3 shows also that there is an excess of studies using subdural ECoG in contrast to a lower number of studies using depth electrodes. Consequently, investigations were restricted to mainly neocortical structures instead of structures like the hippocampi. This may have an impact on the small difference between ripples and fast ripples and on the general result of the meta-analysis. Only one of the studies used intra-operative subdural ECoG (van Klink et al., 2014; see Tables 1, 3), while all the others were based on extraoperative subdural ECoG. This may increase the heterogeneity; but the study with intra-operative recording technique shows a very small effect, so that the inclusion of this study may rather be an underestimation than an overestimation of the aggregated effect.

Fifth, we did not list the types of epilepsy syndromes, because this information was only given in a minor portion of studies. Explicit information was given in one study, where the temporal lobe was affected in 4 out of 13 patients (van't Klooster et al., 2011). Other studies reported results from homogeneous patient groups with various locations of the epileptogenic zone, e.g., epilepsy in tuberous sclerosis (Okanishi et al., 2014) and neocortical epilepsy (Cho et al., 2014). Only recently, Hägelen et al. (2013) reported a significant relation between removal of HFO-generating areas and outcome in patients with temporal lobe epilepsy but no such effect in extra-temporal lobe epilepsies. Therefore, we suggest that future outcome-oriented studies should report whether the epileptogenic zone was assumed to be in the temporal lobe or not, for every patient. Similarly, the etiologies of epilepsy are diverse among the selected studies, and detailed reporting would be warranted. This is especially important since the underlying generation mechanisms of HFOs can differ between etiologies.

Finally, we believe that the main sources of heterogeneity are (i) the use of thresholding when identifying channels with HFOs and (ii) the detection mode. These sources may be seen as moderators, and we addressed them in two subgroup analyses.

### 4.3. Moderators

#### 4.3.1. Thresholding

The question of whether thresholding should be done or not is equivalent to the clinical question: *Should surgical resection include the whole area showing HFOs, or should only areas with a high rate of HFOs be considered to be epileptogenic?* Our results point toward the relevance of a threshold. Indeed, it might be crucial to use a threshold to get a significant relationship between removal of HFO-generating areas and outcome. Jacobs et al. (2010b) examined both, the HFO-occurrence without and with threshold and found that HFOs occurred more frequently in channels that were removed in patients with a better outcome compared to patients with a poor outcome. van Klink et al. (2014) confirmed this result for fast ripples, only. Additionally, Jacobs et al. (2010b) found a difference in the resection ratio between patients, based on channels showing HFOs at any rate. For the meta-analysis, we could only use thresholds when the number of channels above a determined threshold was reported (as reflected in the column *threshold* of Table 1).

The confidence intervals of the overall effect in studies with vs. without thresholding do largely—but not completely—overlap. We can speculate that a slightly higher relation between resection ratio and outcome with thresholding indicates that the technique of thresholding deserves more attention. Various different thresholding techniques were used in the examined studies. It would be of interest to directly compare different thresholding techniques within the same data set in order to determine which has the highest validity. Thresholding may be carried out through the use of a statistical heuristic which is based on standard deviations. Given that only a high rate of HFOs is indicative of the epileptogenic zone, determination of a threshold is both crucial and ambiguous. Does the same strategy for threshold selection apply for all brain regions and for all patients? Are standard deviations a good statistical heuristic?

With respect to our meta-analysis, it would have been advantageous if all the studies which used thresholding had provided information like van't Klooster et al. (2011), whereby both the number of channels being countable above and below a defined threshold can be extracted. Providing this information would shed more light on the rationale behind the thresholds and the effect of thresholding on the data.

#### 4.3.2. Is automated detection better?

One heterogeneity which cannot be characterized by reading the articles is hidden in the process of visual detection of HFOs. Studies with visual detection likely differ from each other because of the subjective nature of visual identification (Von Ellenrieder et al., 2012). Therefore, it is perfectly valid to question whether automated detection yields more reliable results, which have a better relation to outcome.

In our study, the confidence intervals of visual and automated detection effects completely overlapped. The difference between the means of outcome groups is slightly larger for automated than visual detection. We may speculate that automated detection is unlikely to be worse than visual detection. This result should encourage the search for the best automated detection method.

Detection methods vary widely, as does their relation to outcome. In order to find the best automated detector, researchers compared different automated detectors with each other by validating them against the same set of visually detected HFOs (Zelmann et al., 2012; Chaibi et al., 2013a). However, there are no studies comparing the relation between detected HFOs and outcome for the different detection algorithms. All of the automated detectors are developed according to and validated against a ground truth of visually identified HFOs (Staba et al., 2002; Gardner et al., 2007; Kobayashi et al., 2009; Zelmann et al., 2009; Crépon et al., 2010; Jacobs et al., 2010a; Zelmann et al., 2010; Dümpelmann et al., 2012; Salami et al., 2012; Von Ellenrieder et al., 2012; Zelmann et al., 2012; Chaibi et al., 2013a,b; Graef et al., 2013; Pali et al., 2013; Burnos et al., 2014). An unsupervised machine learning approach (Blanco et al., 2010) confirmed the classes of HFO events, but did not evaluate their relevance for the outcome. It would be of interest whether HFOs, as identified by an automated detector that is based on an unsupervised learning approach, are related to outcome.

### 4.4. Publication bias

We used funnel plots and the trim and fill method (Duval and Tweedie, 2000a,b) to estimate what the effect would be like if null or negative results had been suppressed. First, due to asymmetry on the right side of the plot, the method did not succeed in estimating a negative publication bias for ripples and instead only added publications to the right side. It is unlikely that positive results are suppressed and the failure of augmenting the data on the left side does not mean that there is no publication bias. Second, no asymmetry was detected for fast ripples.

The main disadvantage of the trim and fill method is that it is built on the strong assumption that the funnel plot should be symmetric (Sterne et al., 2011). The true mechanism for publication bias is not known and the method performs rather poorly when the between-study heterogeneity is high (Terrin et al., 2003; Peters et al., 2007), which is the case in our study.

Although the results of the trim and fill analysis might be misleading, we want to discuss possible sources of publication bias and related issues. Specifically, the heterogeneity of the methodology in the selected studies suggests that the effect was not detectable without application of methods such as thresholding, or only a certain subtype of HFOs showed a significant relation to outcome (Kerber et al., 2014a). Thus, instead of reporting bias, we should rather think of *outcome reporting bias* (Higgins et al., 2011). This kind of bias is due to the fact that out of a range of outcome measures, the selection of outcomes that are reported can be influenced by the results.

We propose that this shortcoming in the area of HFO research can be addressed by encouraging authors of future outcome-oriented HFO research to comply with a detailed reporting style, which will be outlined in the following text.

### 4.5. Detailed and standardized reporting style

In order to provide the motivation for a more detailed reporting style, we would like to discuss how some of our results appear to contradict the original publications. For example, in the study of Jacobs et al. (2010b) we found a positive mean difference for ripples but not for fast ripples, which is the opposite to what was reported in the original study. The reason for this difference is that we only considered Engel class 1 to be a good outcome (i.e.,: seizure free patient group), while in the original study class 1 and 2 were considered to be a good outcome. The results of Jacobs et al. (2010b) may not be comparable directly with other results when different definitions of good outcome were used. In order to recognize such sources of heterogeneity and to interpret the studies accordingly, it is crucial that researchers report details for each patient. We propose that each outcome-oriented study on HFOs should report the following details:

- outcome class (seizure free vs. not seizure free); we recommend to use ILAE classification
- age
- type of epilepsy
- etiology of epilepsy
- type and frequency of seizures before surgery
- minimum time of follow up
- type(s) of electrodes and exposed surface
- total number of electrodes implanted
- total number of electrodes showing HFOs
- if applicable, total number of electrodes showing HFOs above a defined threshold
- for both of these numbers, the proportion of electrodes that lie in the resected area
- average frequency for ripples and/or, if applicable, fast ripples

and details on the study, including

- if applicable, algorithm for automated detection
- if applicable, algorithm for thresholding
- if applicable, the number of different raters for visual detection (i.e., did one rater detect all the HFOs for all patients? If not, how many people were involved in this task?)
- if applicable, the definition of HFOs used for visual detection
- when ripples and fast ripples are analyzed, the results should be reported separately
- type of EEG that was analyzed (sleep/wake, ictal/interictal…)
- total length of the EEG-segment(s) in which HFOs were detected

This presented checklist may serve more as a proposal than as a recommendation. Giving all of the necessary information to allow calculation of the resection ratio is only one possible common denominator in the search for a generally accepted reporting style. In view of the work published in this area, this might currently be the most objective measure. By proposing that the components of this measure be included in all outcome-oriented HFO studies, we want to open the discussion and the search for other measures which are (i) easy to implement, (ii) comparable between studies, (iii) quantitative, and (iv) objective.

## 5. Conclusion

In this meta-analysis, we were able to show that the relation between removal of HFO-generating brain regions and outcome is significant, but small. This is true for both ripples and fast ripples. There is a considerable between-study heterogeneity, especially with respect to the detection of HFOs and the rating of whether a channel was showing HFOs at all or if the rate of occurrence of HFOs exceeded a certain threshold. Based on the results, we claim that future studies should examine the relation between automatically detected HFOs, thresholding, and outcome. Instead of validating automated HFO detection against a ground truth of visual detection, it should be validated against outcome.

We propose a checklist of details which should be reported when conducting an outcome-oriented HFO study. Complying with these minimal standards should make it easier to compare results with each other, to merge them in meta-analyses, and to objectively assess the true relationship between HFOs and the epileptogenic zone. We absolutely need further examinations which address the question of whether HFOs could be indicative for the epileptogenic zone. Future studies should overcome technical, formal, and methodological shortcomings which may have diminished the significance of previous research.

## Author contributions

YH planned and managed the meta-analysis, extracted the data of the retrieved literature and wrote the paper. PH and AT were co-raters for the literature research. PH additionally contributed to the writing of the introduction and prepared Figures 1, 2. AB, RK, and LK performed the meta-analysis. RK and LK additionally helped with data-extraction. ET, RN, JJ, and AT contributed to the introduction and discussion. In addition, ET and JJ reviewed the whole paper as scientific mentors.

### Conflict of interest statement

The authors declare that the research was conducted in the absence of any commercial or financial relationships that could be construed as a potential conflict of interest.
