# Supplementary material for: High-frequency oscillations in epilepsy and surgical outcome. A meta-analysis
Source: Front Hum Neurosci. 2015 Oct 20;9:574. doi: 10.3389/fnhum.2015.00574 (PMC4611152; doi:10.3389/fnhum.2015.00574)
Supplement: Supplementary file 1 [file Image1.PDF]

Supplementary Material: High-frequency oscillations in epilepsy and surgical outcome. A meta-analysis.

Yvonne Höller [1*], Raoul Kutil [2], Lukas Klaffenböck [2], Aljoscha Thomschewski [1], Peter Höller [1], Arne C Bathke [2], Julia Jacobs [3], Alexandra C Taylor [1] , Raffaele Nardone [1,4] and Eugen Trinka [1]

[1] *Department of Neurology, Christian Doppler Medical Centre and Centre for Cognitive Neuroscience, Paracelsus Medical University, Salzburg, Austria*
[2] *Department of Mathematics, Paris Lodron University, Salzburg, Austria*
[3] *Department of Neuropediatrics and Muscular Diseases and Epilepsy Center; University Medical Center, Freiburg, Germany*
[4] *Department of Neurology, Franz Tappeiner Hospital, Merano, Italy*

Correspondence*:
Yvonne Höller
Department of Neurology, Paracelsus Medical University,
Ignaz-Harrer-Str. 79, 5020 Salzburg, Austria, yvonne.hoeller@pmu.ac.at

1 SUPPLEMENTARY FIGURES

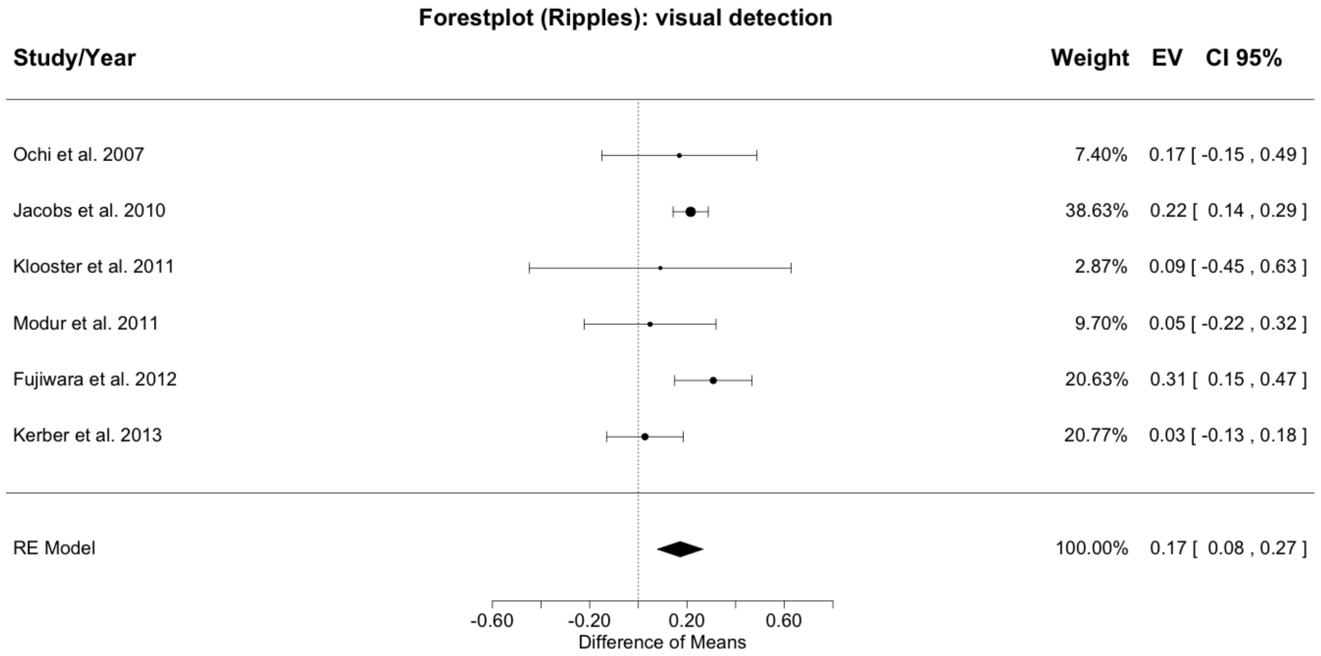

Figure 1. Meta-analysis results for visually detected ripples. The resection ratio is higher in seizure free patients compared to non-seizure free patients. For each study, a graphical representation of the effect (i.e. the difference of the resection ratio between the good- and bad-outcome group) and of the confidence interval (CI) is given along with the exact values (EV) and the weights.

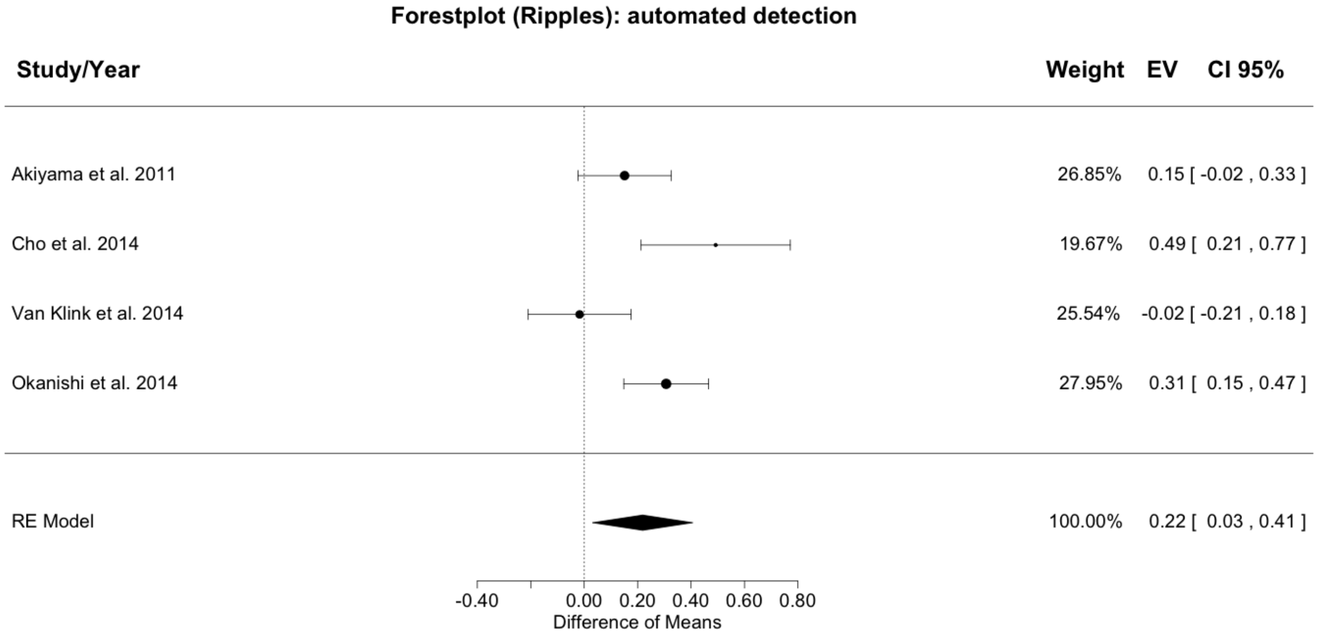

Figure 2. Meta-analysis results for automatically detected ripples. The resection ratio is higher in seizure free patients compared to non-seizure free patients. For each study, a graphical representation of the effect (i.e. the difference of the resection ratio between the good- and bad-outcome group) and of the confidence interval (CI) is given along with the exact values (EV) and the weights.

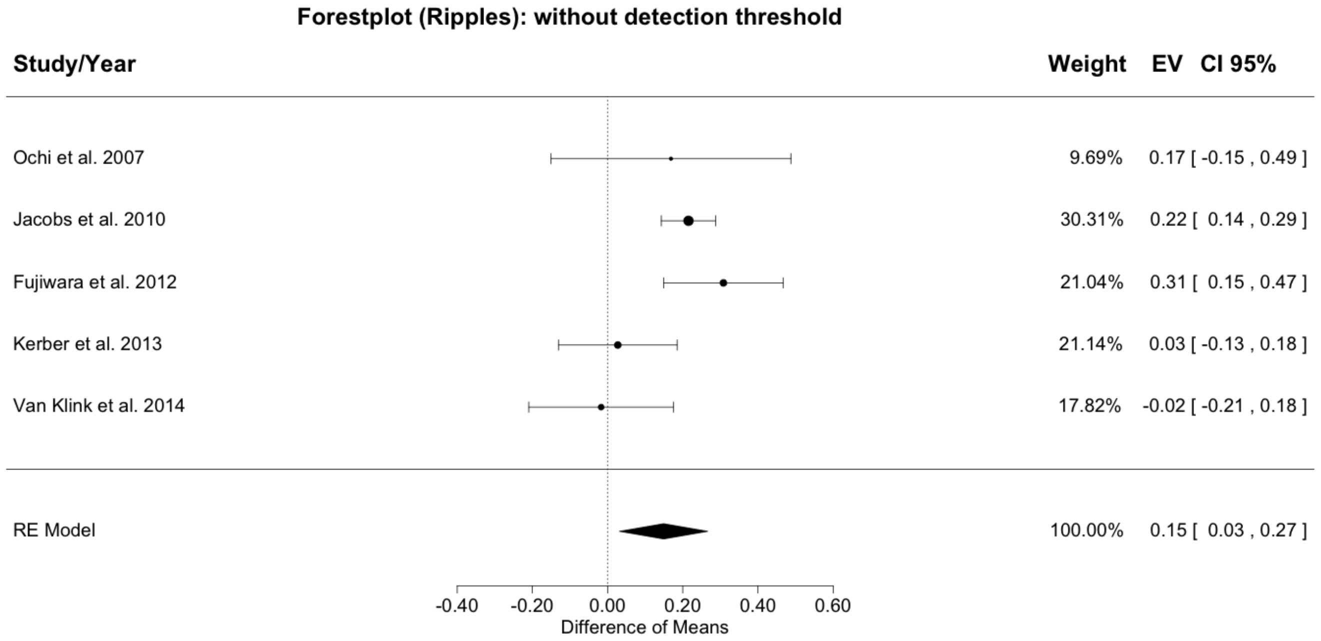

Figure 3. Meta-analysis results for ripples where channels were considered as HFOs regardless the frequency of HFO occurrence. The resection ratio is higher in seizure free patients compared to non-seizure free patients. For each study, a graphical representation of the effect (i.e. the difference of the resection ratio between the good- and bad-outcome group) and of the confidence interval (CI) is given along with the exact values (EV) and the weights.

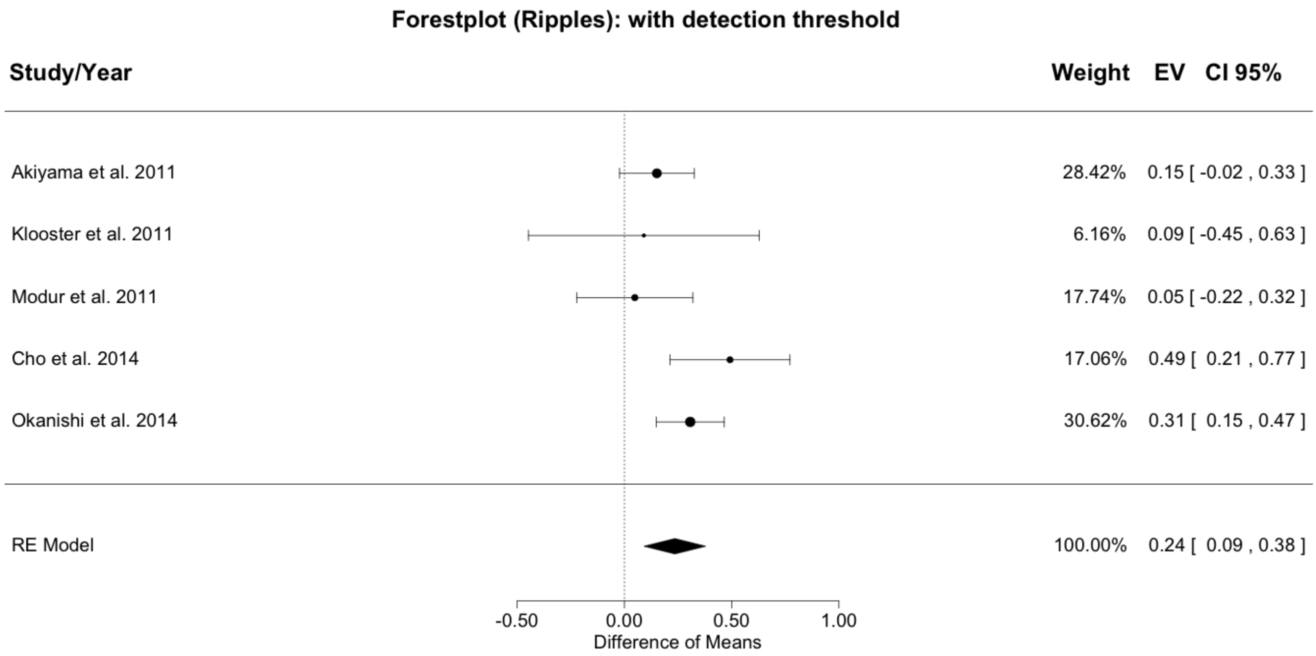

Figure 4. Meta-analysis results for ripples where channels were considered as containing HFOs only if the rate of HFO-occurrence/time interval exceeded a defined threshold. The resection ratio is higher in seizure free patients compared to non-seizure free patients. For each study, a graphical representation of the effect (i.e. the difference of the resection ratio between the good- and bad-outcome group) and of the confidence interval (CI) is given along with the exact values (EV) and the weights.
